# Progression of Early Glaucomatous Damage: Performance of Summary Statistics From Optical Coherence Tomography and Perimetry

**DOI:** 10.1167/tvst.12.3.19

**Published:** 2023-03-20

**Authors:** Emmanouil Tsamis, Sol La Bruna, Anvit Rai, Ari Leshno, Jennifer Grossman, George Cioffi, Jeffrey M. Liebmann, Carlos Gustavo De Moraes, Donald C. Hood

**Affiliations:** 1 Department of Psychology, Columbia University, New York, NY, USA; 2 Albert Einstein College of Medicine, New York, NY, USA; 3 Bernard and Shirlee Brown Glaucoma Research Laboratory, Department of Ophthalmology, Columbia University Irving Medical Center, New York, NY, USA; 4 Sackler Faculty of Medicine, Tel Aviv University, Tel Aviv, Israel

**Keywords:** glaucoma, progression, optical coherence tomography, perimetry, summary statistics, metrics

## Abstract

**Purpose:**

Performance comparison of optical coherence tomography (OCT) and visual field (VF) summary metrics for detecting glaucomatous progression.

**Methods:**

Thirty healthy control eyes (mean deviation [MD], −1.25 ± 2.03; pattern standard deviation [PSD], 1.78 ± 0.77) and 91 patient eyes comprised of 54 glaucoma patients and 37 glaucoma suspects (MD, −1.58 ± 1.96; PSD, 2.82 ± 1.92) with a follow-up of at least 1 year formed a group to evaluate progression with event analyses (P-Event). A subset of eyes with an additional criterion of a minimum of four tests was used for trend analyses (P-Trend) (30 healthy controls and 73 patients). For P-Event analysis, test–retest variability thresholds (lower 5th percentile) were estimated with repeat tests within a 4-month period. A P-Event eye was considered a “progressor” if the difference between follow-up and baseline tests exceeded the variability thresholds. For the P-Trend analysis, rates of change were calculated based on least-squares regression. Negative rates with significant (*P* < 0.05) values were considered progressing. For a reference standard, 17 patient eyes were classified as definitely progressing based on clear evidence of structural and corresponding functional progression.

**Results:**

Isolated OCT and VF summary metrics were either inadequately sensitive or not too specific. Combinations of OCT–OCT and OCT–VF metrics markedly improved specificity to nearly 100%. A novel combination of OCT metrics (circumpapillary retinal nerve fiber layer and ganglion cell layer) showed high precision, with 13 of the 15 statistical progressors confirmed as true positives.

**Conclusions:**

Although relying solely on metrics is not recommended for clinical purposes, in situations requiring very high specificity and precision, combinations of OCT–OCT metrics can be used.

**Translational Relevance:**

All available OCT and VF metrics can miss eyes with progressive glaucomatous damage and/or can falsely identify progression in stable eyes.

## Introduction

Determining whether an eye with glaucoma is progressing is fundamental to the management of glaucoma. Most clinicians rely on measures of visual field (VF) tests based on standard automated perimetry, usually with the 24-2 or the 30-2 test patterns. More recently, measures of optical coherence tomography (OCT) are increasingly being employed, usually to supplement the VF measures. However, which measure of progression to use in particular or, in fact, whether to use VF, OCT, or both are still under debate.

Frequently, global and sectoral summary metrics from OCT and VF tests are used by glaucoma specialists to determine whether an eye shows progressive glaucomatous damage. One example is Zeiss’ Guided Progression Analysis (GPA) software (Carl Zeiss Meditec, Dublin, CA), which was initially developed to identify progression on the 24-2 VF tests. It uses trend analyses to calculate rates of change for the visual field index (VFI) and the mean deviation (MD) summary metrics in an attempt to predict what may happen if the current trend continues unchanged.^1^^,^^2^ More recently, this approach has been extended to include trend- and event-based analyses on averaged global and sectoral circumpapillary retinal nerve fiber layer (cpRNFL) and ganglion cell layer (GCL) measures obtained with OCT.^3^ A number of studies have suggested different thresholds (for event-based analyses) or rates of loss (for trend analyses) for various OCT and VF summary metrics.^4^^–^^9^ For example, in previous studies, series of 24-2 VFs with a rate of 1 dB/y of MD loss have been defined as moderate to fast progression.^5^^,^^8^^,^^10^ In fact, Saunders et al.^10^ concluded that even slower progression rates can be devastating in young patients with advanced loss. Similarly for OCT, it has been suggested as a general rule of thumb that a loss of 5 µm on the global cpRNFL between two OCT scans constitutes strong evidence of progressive glaucomatous loss,^11^^,^^12^ although this measure has been questioned.^13^^,^^14^

However, previous work from our group and others has shown that relying solely on summary metrics from either OCT or VF for the detection of progression can lead to an excessive number of false positives (FPs) and false negatives.^13^^–^^19^ For OCT metrics, the most frequent reasons for these mistakes are segmentation errors and misalignment of OCT scans.^13^^,^^14^^,^^17^^,^^18^ Meanwhile, for VF testing, high test–retest variability affects perimetric measures.^20^^,^^21^ In addition, it is well documented that all summary metrics will miss focal progression due to the averaging that occurs in a given sector/region.^13^^,^^15^^,^^16^^,^^18^ As a result, various guidelines and recommendations have suggested confirmation of progression with repeated testing.^3^^,^^5^^,^^22^ For perimetry, for example, it is accepted that four or more tests are necessary when it comes to calculating the rate of MD or VFI loss.^23^^,^^24^ In clinical reality, this amounts to confidently detecting progression only after 2 or 3 years.

Because there is no clear consensus on the definition of progression based on these summary metrics, it is difficult to evaluate their performance. Not only do inclusion criteria vary across studies, but the definition of what constitutes progression is different, as well. The main purpose of this study was to compare the performance of a variety of commercially available structural and functional metrics on the same set of eyes and against the same definition of both glaucoma and its progression.

## Methods

### Participants

A total of 203 eyes from 166 participants were enrolled for a large observational, prospective, case-control study—the Macular Damage in Early Glaucoma and Progression Study (C. Gustavo De Moraes, PI; ClinicalTrials.gov identifier: NCT02547740). Cases and healthy controls were recruited from the ophthalmology clinics of Columbia University Medical Center/New York Presbyterian. According to the protocol, participants were invited to be tested repeatedly within the first 4 months, then at 6-month and 12-month intervals from baseline and every 6 months thereafter. All participants were required to have at least two study visits to acquire the appropriate OCT scans and VFs. Over 95% of study visits had OCT scans and both 24-2 and 10-2 VFs acquired on the same date. The remaining 5% had a median difference of 9 days between the OCT and VF tests (interquartile range [IQR], 6–27 days; range, 1–5.3 months). Of the 166 participants, 116 were patients with glaucoma (*n* = 73) or were glaucoma suspects (*n* = 43), and 50 were healthy controls (HCs). All HCs had intraocular pressure within normal limits (≤22 mmHg), normal VFs, and normal fundus examination. All patient eyes had a glaucoma or glaucoma suspect diagnosis based on the referring glaucoma specialist's interpretation of functional (24-2 and 10-2 VFs) and structural (fundus photographs, OCT) information, as well as intraocular pressure and clinical history. However, note that the patient diagnosis (i.e., glaucoma patient or suspect) did not play a role in the analyses of this study. In addition, upon recruitment, all eyes had a 24-2 MD better than −6 dB, best-corrected visual acuity better than 20/40, and open angles. Exclusion criteria included significant cataracts, severe myopia or hyperopia (refractive error greater than −6 or +6 diopters, respectively), previous ocular surgery (aside from uncomplicated cataract extraction and/or trabeculectomy, LASIK, or refractive surgeries), other retinal and optic nerve comorbidities (e.g., diabetic retinopathy, macular edema, exudative age-related macular degeneration, geographic atrophy), vein or artery occlusion, amblyopia, and uveitis.

Progression was assessed based on event- and trend-based approaches. For an event-based (P-Event) analysis, a study group (P-Event group) was created based on eyes that had at least one follow-up test a year or more from the first/baseline test. The P-Event group consisted of 121 eyes (30 HCs and 91 patients/suspects) with an average of 33.4 months between the first and last test (range, 12–59 months). Of the 121 eyes, all but 17 had series of four or more tests; eight eyes had two tests, and nine eyes had three tests. For a trend-based (P-Trend) analysis, a criterion of a minimum of four tests was required in addition to the minimum time difference (i.e., 1 year) between the first and last tests. This P-Trend group consisted of 103 eyes (30 HCs and 73 patients/suspects). The average time between the first and last tests was 28 months (range, 12–53 months), and the average number of visits per series was seven (range, 4–13 study visits).

Study procedures followed the tenets of the Declaration of Helsinki and the Health Insurance Portability and Accountability Act and were approved by the Institutional Review Board of Columbia University. Written informed consent was obtained from all participants.

### OCT Data

All eyes were scanned with the SPECTRALIS HRA+OCT (Heidelberg Engineering, Heidelberg, Germany) with the Glaucoma Module Premium Edition (GMPE) protocol. By default, the GMPE protocol allows for the acquisition of a baseline series of scans, and then, at follow-up sessions, the scans are placed in the same location, using SPECTRALIS eye-tracking capabilities.

As part of the GMPE, 24 radial scans were acquired over the optic disc. Based on these radial scans, the average Bruch's membrane opening–minimum rim width (BMO-MRW) was measured for a global (G) and six sectoral summary metrics (see Supplementary Fig. S1, red rectangle). Next, three circumpapillary (circle) OCT scans were obtained centered on the disc with diameters of 3.5, 4.1, and 4.7 mm. From each circle scan, the average cpRNFL thickness was measured for the same seven regions (G and six sectors). For our analyses, we used the summary metrics from the small (3.5-mm) scans (Supplementary Fig. S1, blue rectangle). Finally, the cube scans of the posterior pole were obtained; these were centered on the fovea and obtained along an axis from the foveal center to the BMO center. Again, G and six sectoral metrics were calculated for (1) total retinal, (2) RNFL, (3) GCL (Supplementary Fig. S1, green rectangle), and (4) inner plexiform layer thicknesses. We did not correct any layer segmentation or BMO errors so as to replicate typical clinical practice.

In addition, a novel structure–structure (S-S) metric was calculated based on a combination of OCT summary metrics. This combination is inspired by previous work that highlights the benefits of seeking topographical agreement between GCL and cpRNFL abnormalities.^15^^,^^25^ For an eye to be progressing, it had to show statistical progression in both the macular GCL and cpRNFL regions in the superior and/or the inferior retina. In particular, we defined inferior S-S progression as (1) progression occurring in the temporal–inferior (TI) cpRNFL sector (Supplementary Fig. S2, right panel, where the metrics are highlighted in dark gray) *and* (2) the inferior (I) or the TI GCL sector (Supplementary Fig. S2, left panel, where the metrics are highlighted in dark gray). Here, we abbreviate this inferior S-S agreement as (TI_small_ *and* [TI_GCL_ *or* I_GCL_]). Similarly, a superior S-S progression was defined as progression in the temporal–superior (TS) cpRNFL sector *and* progression in the TS or the superior (S) GCL sector, abbreviated as (TS_small_ *and* [TS_GCL_ *or* S_GCL_]) (Supplementary Fig. S2, metrics highlighted in dark red). Finally, a progressing eye was defined as an eye that showed inferior S-S progression *or* superior S-S progression (or, as we refer to it, the S-S metric).

### VF Data

All eyes had VF testing with a Humphrey Field Analyzer (HFA; Carl Zeiss Meditec), using the 24-2 and the 10-2 testing patterns (random order of tests, Swedish Interactive Threshold Algorithm standard strategy). The MD, pattern standard deviation (PSD), and VFI were obtained from the 24-2 single field report, and the MD and PSD from the 10-2 report were used (see Supplementary Fig. S3; green and red rectangles, respectively). VF tests were excluded if FP responses were greater than 15% or fixation losses were greater than 33%.^26^ Apart from the global indices described above, many perimetric devices also perform analyses of progression on clusters. Although Octopus 9000 (Haag-Streit, Köniz, Switzerland) reports on cluster analysis are commercially available, the HFA provides only the superior MD (supMD) and inferior MD (infMD) metrics of the 24-2 and 10-2 VFs via the Zeiss FORUM platform. As such, we used the “Advanced Data Export” module from FORUM to export the supMD and infMD metrics of the 24-2 and 10-2 VFs. Although these metrics may not be readily available to every glaucoma practice and/or research center that uses an HFA, we exported and analyzed their performance to test the hypothesis that sectorial VF metrics perform better than their global counterparts.

### Identifying Statistical Progressors

For the P-Event analysis, we estimated test–retest (short-term) variability from any eligible eyes that had at least two OCT and two VF tests within 4 months.^27^^,^^28^ From the initial dataset of 203 eyes, 176 eyes (146 patients/suspects, 30 HCs) fulfilled those criteria (median number of tests, 4; IQR, 2–4). The repeated OCT and VF data were analyzed with quantile regression to define thresholds and cut-offs.^18^ The baseline values from the first test in the series were considered the independent variable, and follow-up measures formed the dependent variable. The final test–retest dataset consisted of over 2100 pairs of measurements. Of these ∼2100 pairs, only 135 had a time difference of more than 90 days (i.e., the pair of tests was between 3 and 4 months apart); approximately 1700 pairs (>80%) were within 60 days (2 months). Statistical progression was defined and evaluated at two cut-off criteria: the 5th (one-tail significance) and the 2.5th percentile (two-tail significance; 95% confidence interval [CI]). Those cut-offs were then applied to the first versus last test of the P-Event group in order to identify eyes with statistical progression.

For a trend-based analysis, all tests within each series of the 103 P-Trend eyes were used. Based on ordinary least squares regression, eyes were categorized as statistical progressors if the slope was significantly negative. Two significant one-tail *P* levels were evaluated: *P* < 0.05 and *P* < 0.025. Note that the *P* < 0.01 criterion was also evaluated; however, most summary metrics were highly specific (>98%), thus making it difficult to draw significant conclusions with regard to which summary metrics perform the best at that criterion.

### Performance Analysis

**Figure 1.**
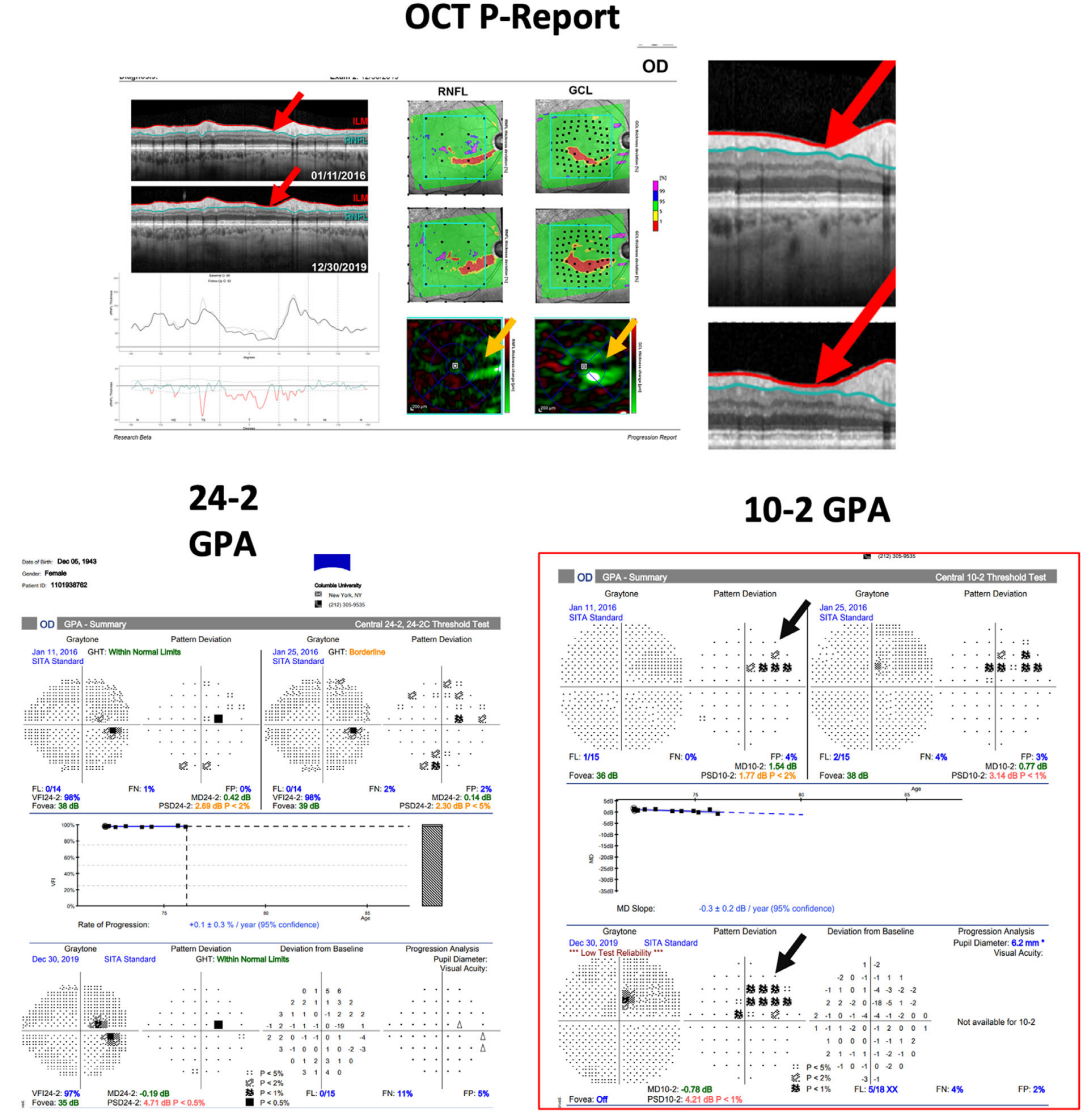
An example of a definitely progressing (DP) eye with the one-page Progress OCT Report (top) and the corresponding VF 24-2 and 10-2 Guided Progression Analysis (GPA) reports (bottom). *Red arrows* highlight the changes/loss of retinal nerve fiber layer (RNFL) on the circumpapillary b-scans (also enlarged). *Orange arrows* indicate the areas of thinning in the RNFL and ganglion cell layer (GCL), as shown in the relevant change maps. *Black arrows* on the 10-2 GPA highlight the area of sensitivity reduction in the 10-2 visual field.

For each metric, specificity was estimated based on the number of HC eyes that were falsely identified as statistically progressing; these were clear FPs. However, calculating sensitivity is complicated due to the lack of an accepted reference standard and the lack of a single test that confirms progression of glaucomatous damage. Just because an eye is labeled as progressing based on a particular metric that falls outside the 95% CI does not mean that eye is truly progressing. To identify FPs, we evaluated all eyes labeled as statistically progressing by any metric following a previously described approach. In particular, we used a combination of OCT and VF information to validate progression. This method used a newly developed OCT progression (OCT-P) report, introduced by Hood et al.,^29^ that allows for structural information from the first and last test to be presented on a one-page report. In addition, we used the total and pattern deviation probability maps from the corresponding 10-2 and 24-2 VFs to evaluate topographic (structure-function) agreement. Figure 1 shows an example of a progressing eye with the one-page ‘Progress OCT Report’ (top) and the corresponding VF 24-2 and 10-2 GPA reports (bottom).

Based on evidence from these three reports, progression was defined based on three criteria: (1) Is there progressive thinning of the cpRNFL visible on the b-scan images and/or on the cpRNFL difference plot? (2) Are this progressive thinning of the cpRNFL and its location confirmed on the RNFL and GCL probability maps and/or on the RNFL and GCL change maps? (3) Is there evidence of a progressive change at corresponding locations of 24-2 and/or 10-2 VFs? If, for a study eye, the answers were positive for all three questions, based on the independent judgment of three glaucoma experts, then that eye was considered to be definitely progressing (DP). It is worth highlighting that the glaucoma experts were masked to the results of the analysis for the summary metrics and whether an eye was marked as progressing by one or more summary metrics. For example, the eye shown in Figure 1 is a DP eye. For this eye, the answer to the first question was “yes,” and the evidence is highlighted with red arrows on the circumpapillary b-scans (also enlarged). For questions 2 and 3, the answers were also “yes,” given the strong evidence of change on the RNFL and GCL change maps (orange arrows), as well as the 10-2 VFs (black arrows).

Through this process, we identified 17 DP patient eyes. Note that this analysis does not exclude the possibility of progression on the other non-DP eyes. We chose to use the 17 eyes that showed clear signs of corresponding progressive damage on both OCT and VF probability maps to reduce the likelihood of a false positive by our reference standard. As such, we can be reasonably confident that progression did indeed occur in the 17 DP eyes. Therefore, these eyes should not be missed by the summary metrics or any other method. In addition to the specificity and sensitivity, we also report on the 95% CIs of each performance measure, as calculated by a bootstrapping method (1000 iterations, samples of equal size drawn with replacement).

## Results

### OCT Summary Metrics

Table 1 shows the number of statistically progressing P-Event eyes based on an event analysis for the most common OCT metrics (i.e., cpRNFL of the 3.5-mm circle, BMO-MRW, and GCL). The number of statistically progressing eyes is shown separately for the HC group (column 2) and the patient group (column 3). For example, for the G metric for cpRNFL thickness based on the 3.5-mm circle scan (G_small_) (Table 1), only one of the HC eyes was falsely identified as progressing, resulting in an estimated specificity of 97% (Table 1). In addition, there were 23 patient eyes (Table 1) that showed statistical progression based on the test–retest variability threshold of the G_small_ metric. Column 4 in Table 1 indicates the sensitivity of each metric based on the number of DP eyes correctly identified. For example, of the 23 patient eyes that the G_small_ marked as progressing, 11 were DP eyes. In other words, we can be reasonably confident that at least 11 of the 23 patient eyes were true positives (TPs). In addition, we are confident that the G_small_ metric missed some eyes with clear progression (in particular, six of the 17 DP eyes), thus achieving a sensitivity of 65% (Table 1). Note that here we present the results for the strictest cut-off criterion (2.5th percentile). Full results for all evaluated metrics for the 5th percentile criterion, which was more sensitive but did not reach adequate specificity, are provided in Supplementary Tables S1 to S4.

**Table 1.** Number of Statistical Progressors at the 2.5th Percentile Cut-Off Level^*^

	30 HCs FP (Specificity [%], 95% CI)	All 91 Patients	17 DPs TP (Sensitivity [%], 95% CI)
cpRNFL Metrics—Small Circle Scan (3.5 mm)			
G_small_	1 (97, 90–100)	23	11 (65, 41–88)
T_small_	1 (97, 90–100)	16	7 (41, 18–65)
TI_small_	2 (93, 83–100)	24	13 (77, 53–94)
TS_small_	0 (100, 100–100)	18	13 (77, 53–94)
N_small_	1 (97, 90–100)	12	5 (29, 12–53)
NI_small_	1 (97, 90–100)	16	10 (59, 35–82)
NS_small_	2 (93, 83–100)	17	11 (65, 41–88)
BMO-MRW Metrics—Radial Scans			
G_MRW_	6 (80, 67–93)	19	11 (65, 41–88)
T_MRW_	3 (90, 77–100)	12	4 (24, 6–47)
TI_MRW_	3 (90, 77–100)	10	7 (41, 18–65)
TS_MRW_	1 (97, 90–100)	14	9 (53, 29–77)
N_MRW_	1 (97, 90–100)	10	6 (35, 12–59)
NI_MRW_	2 (93, 83–100)	9	7 (41, 18–65)
NS_MRW_	0 (100, 100–100)	11	8 (47, 24–71)
GCL Metrics—Posterior Pole Cube Scan			
G_GCL_	1 (97, 90–100)	31	14 (82, 65–100)
I_GCL_	0 (100, 100–100)	13	9 (53, 29–77)
TI_GCL_	0 (100, 100–100)	16	13 (77, 53–94)
NI_GCL_	4 (87, 73–97)	31	11 (65, 41–88)
S_GCL_	1 (97, 90–100)	6	5 (29, 12–53)
TS_GCL_	0 (100, 100–100)	16	6 (35, 12–59)
NS_GCL_	2 (93, 83–100)	16	6 (35, 12–59)

* As defined by event analysis on OCT summary metrics.

Temporal (T), Nasal (N), Nasal Inferior (NI), Nasal Superior (NS), Minimum Rim Width (MRW), Ganglion Cell Layer (GCL).

#### Specificity

All but one of the OCT summary metrics for cpRNFL and GCL thicknesses showed a specificity of ∼95% (i.e., one or two FP HCs). The specificity of the BMO-MRW metrics was not as good. For example, the G_MRW_ falsely identified six HC eyes as progressing.

#### Identifying DP

None of the OCT summary metrics, when used in isolation, was able to detect more than 14 of the 17 DP eyes. The commonly used G_small_ metric missed six DP eyes. The best performing metrics were the TS sector of the cpRNFL (TS_small_) and the TI of the GCL (TI_GCL_) with no FPs (i.e., 100% specificity) and 13 TPs, or a sensitivity of 77% of the 17 DP eyes (Table 1). It is also worth noting the performance of the G_GCL_, which showed the highest number of TPs (*n* = 14) and one FP (i.e., 97% specificity).

### VF Summary Metrics

Table 2 provides the results for the VF summary metrics in the same format as Table 1.

**Table 2.** Number of Statistical Progressors at the 2.5th Percentile Cut-Off Level^*^

	30 HCs FP (Specificity [%], 95% CI)	All 91 Patients	17 DPs TP (Sensitivity [%], 95% CI)
24-2 VF			
MD 24-2	1 (97, 90–100)	21	9 (53, 29–77)
supMD 24-2	1 (97, 90–100)	13	7 (41, 18–65)
infMD 24-2	0 (100, 100–100)	9	5 (29, 12–53)
PSD 24-2	2 (93, 83–100)	7	4 (24, 6–47)
VFI 24-2	2 (93, 83–100)	9	4 (24, 6–47)
10-2 VF			
MD 10-2	1 (97, 90–100)	13	7 (41, 18–65)
supMD 10-2	1 (97, 90–100)	9	5 (29, 12–53)
infMD 10-2	0 (100, 100–100)	7	4 (24, 6–47)
PSD 10-2	3 (90, 77–100)	8	5 (29, 12–53)

* As defined by event analysis on VF summary metrics.

#### Specificity

The specificity of the VF summary metrics was comparable to that of the OCT metrics. The number of FPs ranged from one HC eye for the MDs of the 24-2 and 10-2 VFs to three eyes for the PSD of the 10-2 VF.

#### Identifying DP

All VF metrics showed a significantly lower number of TPs as compared to the OCT metrics. The best performing metrics were the MDs for the 24-2 and 10-2 VFs, which identified nine and seven DP eyes, respectively.

### S-S Combinations

Table 3 pairs commonly used summary metrics for the cpRNFL thickness with BMO-MRW measures (Table 3A) and with GCL thicknesses measures (Table 3B). In particular, we utilized global G metrics as well as the TI and TS sectoral metrics. We evaluated combinations with *and* and *or* operators along with our new metrics, Inferior S-S, Superior S-S, and their combination, the S-S metric.

**Table 3.** Number of Statistical Progressors at the 2.5th Percentile Cut-Off Level^*^

	30 HCsFP (Specificity [%], 95% CI)	All 91 Patients	17 DPsTP (Sensitivity [%], 95% CI)
A. cpRNFL (3.5 mm) and BMO-MRW			
G_small_ *or* G_MRW_	6 (80, 67–93)	30	15 (88, 71–100)
G_small_ *and* G_MRW_	1 (97, 90–100)	12	7 (41, 18–65)
TI_small_ *or* TI_MRW_	5 (83, 70–97)	28	14 (82, 65–100)
TI_small_ *and* TI_MRW_	0 (100, 100–100)	6	6 (35, 12–59)
TS_small_ *or* TS_MRW_	1 (97, 90–100)	22	13 (77, 53–94)
TS_small_ *and* TS_MRW_	0 (100, 100–100)	10	9 (53, 29–77)
B. cpRNFL (3.5 mm) and GCL			
G *and* G_GCL_	0 (100, 100–100)	16	10 (59, 35–82)
G *or* G_GCL_	2 (93, 83–100)	38	15 (88, 71–100)
TI *and* (TI_GCL_ *or* I_GCL_) (Inferior S-S)	0 (100, 100–100)	13	12 (71, 47–88)
TI *or* (TI_GCL_ *or* I_GCL_)	2 (93, 83–100)	30	15 (88, 71–100)
TS *and* (TS_GCL_ *or* S_GCL_) (Superior S-S)	0 (100, 100–100)	7	6 (35, 12–59)
TS *or* (TS_GCL_ *or* S_GCL_)	1 (97, 90–100)	29	15 (88, 71–100)
(Inferior S-S) *or* (Superior S-S)	0 (100, 100–100)	15	13 (77, 53–94)

* As defined by event analysis on combinations of OCT–OCT summary metrics.

#### Specificity

As expected, all combinations with *and* were highly (100%) specific. Note, for example, that the (G_small_ *and* G_GCL_) metric correctly identified all HC eyes as non-progressing. The same was true for our new metrics.

#### Identifying DP

Unsurprisingly, combinations with the *or* operator detected the highest number of DP eyes, as 15 out of 17 DP eyes were correctly identified by four different metrics. In any case, it is worth noting that even the most sensitive of metric combinations will still miss DP eyes, which are eyes with clear glaucomatous progression. Of those metrics with no FPs (i.e., 100% specificity), our new S-S metric (Inferior S-S *or* Superior S-S) detected the highest number of DP eyes (i.e., 13 TPs, for a sensitivity of 77%) (Table 3).

### S-F Combinations

The evaluation of pairings between structural and functional metrics is shown in Table 4. The upper six rows of Table 4 show the commonly used metrics such as the MDs for the 10-2 and 24-2 VFs and the global G metrics for cpRNFL (of the 3.5-mm circle scan) and the GCL. The last two rows provide the results for a combination of our new Inferior S-S *or* Superior S-S metric and the 24-2 and 10-2 global and sectoral MDs. For the combination of the Inferior S-S *or* Superior S-S metric with the sectoral MD metrics (secMD) of the 24- and 10-2 VFs, the requirement was that there was agreement, on a hemifield level, between the structural and functional metrics. That is, both the Inferior S-S metrics *and* the infMD of either the 24-2 or the 10-2 showed progression; similarly, both the Superior S-S *and* the supMD of either the 24-2 or the 10-2 agreed on the presence of progressive damage.

**Table 4.** Number of Statistical Progressors at the 2.5th Percentile Cut-Off Level^*^

Structure and Function	30 HCs FP (Specificity [%], 95% CI)	All 91 Patients	17 DPs TP (Sensitivity [%], 95% CI)
G_small_ *and* MD 24-2	0 (100, 100–100)	9	6 (35, 12–59)
G *or* MD 24-2	2 (93, 83–100)	35	14 (82, 65–100)
G_GCL_ *and* MD 10-2	0 (100, 100–100)	9	7 (41, 18–65)
G_GCL_ *or* MD 10-2	2 (93, 83–100)	35	14 (82, 65–100)
(G_small_ *or* G_GCL_) *and* (MD_24_ *or* MD_10_)	0 (100, 100–100)	13	10 (59, 35–82)
(G_small_ *and* G_GCL_) *and* (MD_24_ *and* MD_10_)	0 (100, 100–100)	6	4 (24, 6–47)
(Inferior S-S) *or* (Superior S-S) *and* (MD_24_ *or* MD_10_)	0 (100, 100–100)	10	10 (59, 35–82)
(Inferior S-S) *or* (Superior S-S) *and* (secMD_24_ *or* secMD_10_)	0 (100, 100–100)	11	11 (65, 41–88)

* As defined by event analysis on combinations of OCT–VF summary metrics.

#### Specificity

The specificity of metrics for structure–function (SF) agreement was comparable to that for S-S agreement. Combinations with the *and* operator had 100% specificity, whereas the *or* combinations were less specific with 2 FPs.

#### Identifying DP

The number of DP eyes detected was, generally, lower than those identified correctly by S-S combinations. The largest number of TPs was 11, as shown by the combination of our new metric and its sectoral functional counterpart: ([S-S metric] *and* [secMD24 *or* secMD10]). Other notable metrics, with 10 TPs, are one that included the global G metrics of cpRNFL and GCL and the 24-2 and 10-2 MDs—([G_small_ *or* G_GCL_] *and* [MD_24_ *or* MD_10_])—and the combination of the S-S metric with the global MDs—([S-S metric] *and* [MD_24_ *or* MD_10_]). However, note that all of the 11 and 10 statistically progressing eyes identified by our new S-F metrics were confirmed as TPs (Table 4), indicating a potentially higher precision.

### Trend Analysis

Defining progression based on trend analysis resulted in a higher number of FPs. A comparison of FPs between trend- and event-based analyses for the most commonly used and best performing summary metrics is shown in Table 5. The number of correctly identified DP eyes was also generally lower than event-based results. The full results of the trend analysis for all OCT and VF metrics and their combinations are provided in Supplementary Tables S5 to S8.

**Table 5.** Comparison of the Number of FPs Between Trend- and Event-Based Analyses

	2.5th Percentile	
	30 HCs FP (Specificity [%], 95% CI) Trend Analysis	30 HCs FP (Specificity [%], 95% CI) Event Analysis
OCT Metrics		
G_small_	5 (83, 70–97)	1 (97, 90–100)
G_MRW_	6 (80, 67–93)	6 (80, 67–93)
TS_small_	1 (97, 90–100)	0 (100, 100–100)
TI_GCL_	2 (93, 83–100)	0 (100, 100–100)
VF Metrics		
MD 24-2	3 (90, 77–100)	1 (97, 90–100)
MD 10-2	3 (90, 77–100)	1 (97, 90–100)
Structure–Structure Combinations		
G_small_ *and* G_GCL_	1 (97, 90–100)	0 (100, 100–100)
[Inferior S-S] *or* [Superior S-S]	0 (100, 100–100)	0 (100, 100–100)
Structure–Function Combinations		
[G_small_ *or* G_GCL_] *and* [MD_24_ *or* MD_10_]	2 (93, 83–100)	0 (100, 100–100)
[Inferior S-S] *or* [Superior S-S] *and* [secMD_24_ *or* secMD_10_]	1 (97, 90–100)	0 (100, 100–100)

### Best Performing Metrics

**Figure 2.**
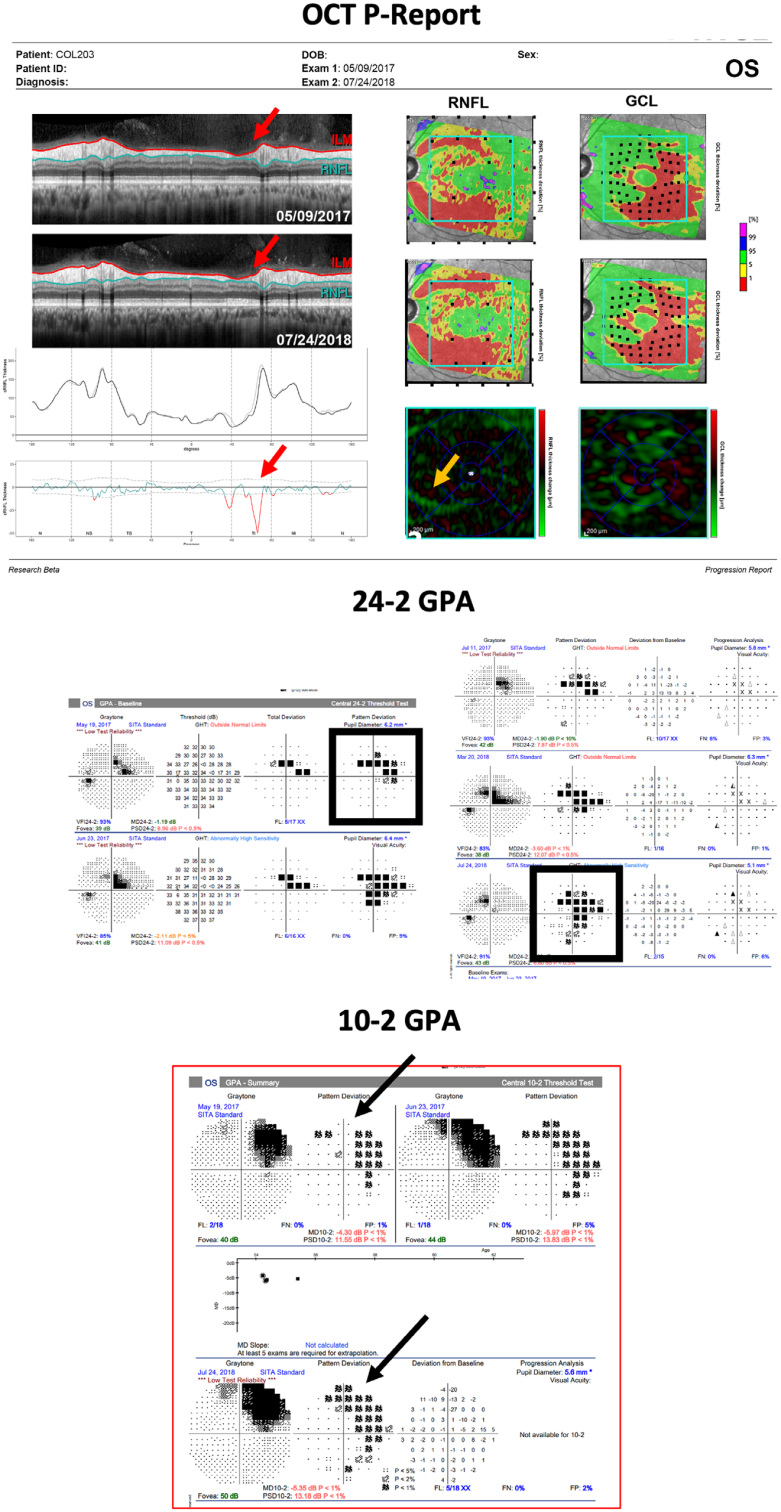
The one-page Progress OCT Report (*top*), the 24-2 VF GPA report (*middle*), and the 10-2 VF GPA report (*bottom*) of the only eye that was missed by all three metrics (i.e., TS_small_, TI_GCL_, and S-S metrics). This eye had localized progressive damage in the inferior retina and disc. *Red arrows* highlight the region of cpRNFL progression on the OCT-P report. *Orange arrows* highlight the corresponding region of change on the RNFL change map. *Black rectangles* (24-2) and *black arrows* (10-2) highlight the progression on the visual fields.

Because the base rate of glaucoma is relatively low, specificity should be as high as possible. Therefore, we considered summary metrics (and combinations) to have excellent performance if they had no FPs (i.e., 100% specificity) and good sensitivity (i.e., a high number of correctly identified DP eyes). Based on these criteria, we identified three summary metrics as the best performers: TS_small_, TI_GCL_, and S-S. Each of these three metrics correctly identified progression in 13 of the 17 DP eyes (77%). Of the 17 eyes, 10 eyes (or 59%) showed progression in all three metrics, and all but one DP eye showed progression based on at least one metric. Figure 2 shows the only eye that was missed by all three metrics. This eye had localized progressive damage in the inferior retina and disc. The cpRNFL region of progression is highlighted with a red arrow on the OCT-P report, and the corresponding region on the RNFL change map is noted with orange arrows on the same report. This eye also had progressing damage on the superior VF, noticeable on the 24-2 (black rectangles) and the 10-2 (black arrows) VFs.

## Discussion

Identifying the progression of glaucoma is of major importance in the clinic. Numerous studies have evaluated different summary metrics readily available from OCT and visual field instruments.^1^^–^^3^^,^^5^^–^^7^^,^^9^^–^^11^ It is difficult, however, to draw safe conclusions about the optimal metrics to use from the results of these studies, as most of them evaluate different sets of metrics, use different reference standards, and employ different inclusion criteria. In this study, we used OCT and VF tests to evaluate the performance (i.e., sensitivity and specificity) of commercially available and routinely used summary metrics in detecting progression of glaucomatous damage. In addition, we have reported on the effect upon these performance measures when summary metrics are combined, based either on the same modality (e.g., BMO and cpRNFL metrics from OCT scans) or on OCT-VF pairings. For the evaluation and analyses of sensitivity and specificity, we attempted to avoid including eyes whose diagnosis of progression was uncertain. Therefore, for specificity measures we relied on 30 HCs, whereas for the calculation of sensitivity we evaluated 17 glaucomatous eyes where progression was evident, beyond reasonable doubt, on both OCT and VF tests.

### Single Metrics Make Mistakes

We found that all OCT and VF summary metrics, when used in isolation, will miss some glaucomatous eyes with clear evidence of progressive loss. For example, all 24-2 and 10-2 VF summary metrics missed progression, shown in both OCT and VF probability maps, in at least half of the total 17 DP eyes. Some of the best performing OCT summary metrics (e.g., TS_small_, G_GCL_) performed slightly better. Both the TS_small_ and the G_GCL_ metrics correctly identified 13 and 14 DP eyes, respectively, for a sensitivity of approximately 80%. In addition, most OCT and VF metrics will wrongly identify progression in HC eyes. In particular, BMO-MRW summary metrics from radial disc scans were prone to FPs, with the global metric G_BMO_ making six mistakes. On the other hand, five of the OCT summary metrics (Table 1) reached a specificity of 100%, but none of the VF metrics achieved that level of specificity (Table 2).

### Combining Summary Metrics

The results of this study agree with previous evidence concerning the benefits of topographic agreement, either between measures of the same modality (e.g., OCT and cpRNFL-GCL pairings) or OCT-VF combinations.^15^^,^^16^^,^^25^^,^^30^ Most combinations of the summary metrics shown in Tables 4 and 5 had a specificity between 97% and 100%. However, OCT-VF combinations did not perform as well as their structural counterparts. For example, the best performing S-F metric had a sensitivity of 65%.

In comparison, our new S-S metric had a sensitivity of 77%, with 100% specificity. Another interesting feature of the S-S metric is its high precision level at 87%. In other words, of the 15 statistically progressing eyes, 13 were confirmed as true positives. This indicates that, if an eye shows progression based on the S-S metric, then it is likely that this eye is a true progressor.

### Event VS Trend Analysis

A unique feature of this study is the comparison of both event- and trend-based analyses. We found that an event-based approach, which compares the most recent test against a baseline, performs better than trend analysis of long test series. Specificity measures were lower for the majority of the OCT and VF metrics and their combinations when trend analysis was performed. One reason for this reduced performance may be the relatively short duration in the series of tests as compared to previous studies,^22^ with the average time between the first and last test in our study group being approximately 2.5 years. On the other hand, there are several disadvantages in using an approach that compares first and last test (i.e., event-based). For example, scan artifacts and segmentation errors for OCT and a patient's variability and learning effects for VFs are significant contributors to false categorizations (i.e., both false negatives and false positives). Event-based analyses could potentially be improved if we evaluate more than one pair of tests in order to confirm our initial classification. In this study, we also evaluated the performance of comparisons between second baseline and last tests (second vs. last test). The sensitivity and specificity of those comparisons were very similar (almost identical) to the first versus last test approach (data not shown). Regardless, it is likely in the patient's best interest for a clinician to be able to detect signs of glaucomatous progression since the patient's last visit (i.e., an event-based approach) rather than waiting for a longer time period.

### Clinical Relevance

Although clinicians rarely rely on one summary metric, their final decisions are often informed by previously suggested thresholds and guidelines—for example, a rate of MD loss higher than 1 dB/y,^5^^,^^8^^,^^10^ a change in the global cpRNFL metric of more than 5 µm between two tests,^6^^,^^7^^,^^11^^,^^14^ or a significant negative trend on the VFI index,^1^^,^^2^ among others.^23^^,^^24^ The results of this study indicate that none of the summary metrics can solely be trusted for the detection of progressive glaucomatous damage, and careful evaluation of the entire OCT and VF reports is needed to make that decision. Indeed, we have previously shown that focusing on topographical patterns and seeking agreement between VF and OCT tests can be more efficient in detecting glaucomatous progression than the use of summary metrics.^15^^,^^16^^,^^25^^,^^31^ However, here we propose that some of these metrics, such as the S-S metric, could have an important role as auxiliary tools in clinical decision making, given the high specificity and adequate sensitivity and precision that they present. They can be used, for example, to guide clinicians in prognosis and management in addition to early detection of progression. Such metrics may be useful in triage clinics to decide which patients need to see a glaucoma specialist/surgeon imminently or whether they can be assessed virtually.

### Implications for Clinical Trials

Although summary metrics are not optimal for clinical purposes, there is a potential for using some as objective endpoints in clinical trials investigating glaucomatous progression. Based on our results, the highly specific and precise S-S metric and/or its combination with the VF sectoral MD (last row, Table 4) are the most promising candidates. Alternatively, these summary metrics can be part of the inclusion criteria when recruitment procedures require eyes with clear glaucomatous progression.

### Limitations

All eyes included in this study had an MD measure at baseline 24-2 VF better than –6 dB. As a result, the test–retest variability cut-off limits that were determined in this study are more representative of cases with early defects or without defects. It is, therefore, possible that the conclusions would differ for eyes with more advanced disease, where measurement noise is likely larger, and the dynamic range of summary metrics based on standard automated perimetry may be greater than the dynamic range of OCT metrics. In addition, pointwise or region-of-interest measures and analyses would hold a significant advantage over summary measures in cases where most of the OCT (thickness) or the VF (deviation) maps are at their lower limits.^32^^–^^35^

In addition, the fact that we did not manually correct the segmentation of scans might have affected the detection performance of OCT metrics, which could be considered a limitation for this study. This approach might have affected the results of the BMO-MRW metrics, where we found that the automated algorithm would frequently fail to detect the appropriate BMO point, especially in the presence of vessels. Nonetheless, we opted not to apply any changes to the automated measures so as to be more representative of real-life clinical practice.

For a trend-based evaluation of the 24-2 and 10-2 VF metrics, we performed ordinary least squares regression analysis on all available VF metrics. This approach assumes some linearity in the reduction of the summary metrics across time, an assumption that does not strictly apply to the PSD metric or probably the VFI metric, as well. Nevertheless, given the relatively small duration of the test series (average time between first and last test was 22 months), the effect of such an assumption is likely minimal.

It is worth noting that some VF summary metrics were adjusted for age (e.g., the 24-2 MD) whereas structural thickness measurements were not. The implications of this characteristic when comparing OCT and VF summary metrics for evaluating progression are unclear. If, indeed, age adjustment was a factor, then we would expect unadjusted metrics to produce more FPs in the HC group than the age-adjusted metrics. The results of our study do not support this hypothesis; however, the average time between first and last tests was only 2.3 years. It is, therefore, unlikely that aging was a major factor during our short study period.

Finally, another limitation is the relatively small number of DP eyes. Evaluating sensitivity measures based on only 17 eyes is certainly not ideal; however, the purpose of this study was to use patient eyes with clear evidence of progression in both the OCT and the VF. As such, one would expect the summary metrics and, in fact, any other method to correctly identify all of the 17 DP eyes. Any inclusion of additional eyes that showed progression on either the OCT or the VF would introduce a bias toward one test or another, whereas it would also increase the likelihood of FPs (i.e., progression) by the reference standard. Although an evaluation of sensitivity with a larger sample of eyes over longer follow-ups and in patients with varying degrees of glaucoma severity would provide a better estimate of the true performance of OCT and VF summary metrics, our major conclusion would nonetheless be the same: *All* *summary metrics will miss some eyes with clear progressing glaucomatous damage.*

## Conclusions

A method for evaluating progression that relies solely on the use of single or combined OCT and VF metrics can fail to identify eyes with clear progression, shown on OCT and VF maps, and will often falsely categorize some HC eyes as progressing. However, some combinations of OCT metrics (S-S) and OCT-VF pairings can be highly specific, a feature that can prove to be useful for triage purposes and clinical trials. Nonetheless, clinicians should not rely solely on OCT and/or VF summary metrics for the detection of progressive glaucomatous damage. Instead, they should be able to make better decisions about progression between two test visits if they use a method similar to the reference standard in this study^15^^,^^16^—specifically, a topographic evaluation of changes on OCT b-scan images and thickness and probability maps, as well as a topographical comparison to changes seen on OCT and VF deviation/probability maps.

## Supplementary Material

Supplement 1

Supplement 2

Supplement 3

Supplement 4

Supplement 5

Supplement 6

Supplement 7

Supplement 8

Supplement 9

Supplement 10

Supplement 11
