# Supplementary material for: Progression of Early Glaucomatous Damage: Performance of Summary Statistics From Optical Coherence Tomography and Perimetry
Source: Transl Vis Sci Technol. 2023 Mar 20;12(3):19. doi: 10.1167/tvst.12.3.19 (PMC10043504; doi:10.1167/tvst.12.3.19)
Supplement: Supplement 1 [file tvst-12-3-19_s001.pdf]

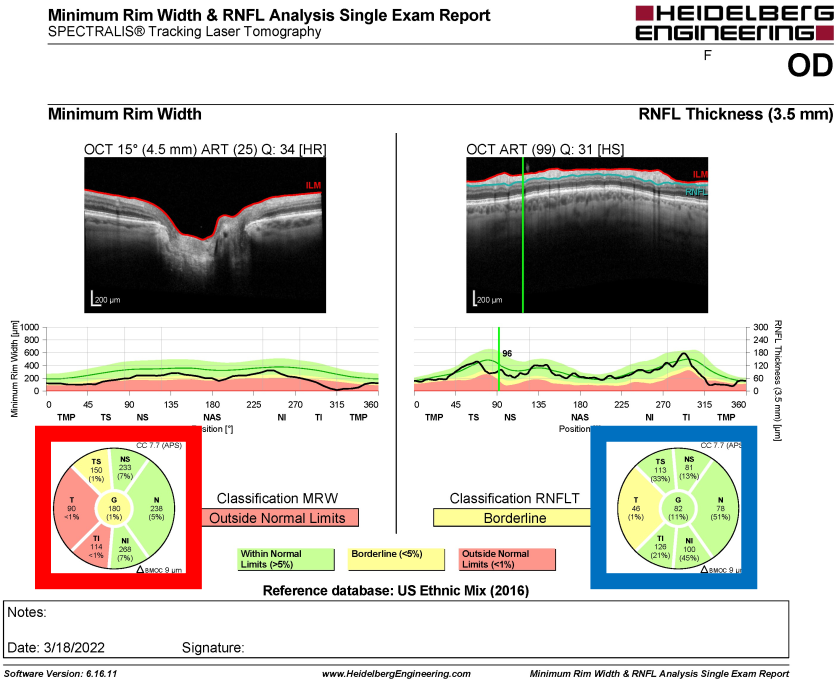

GCL

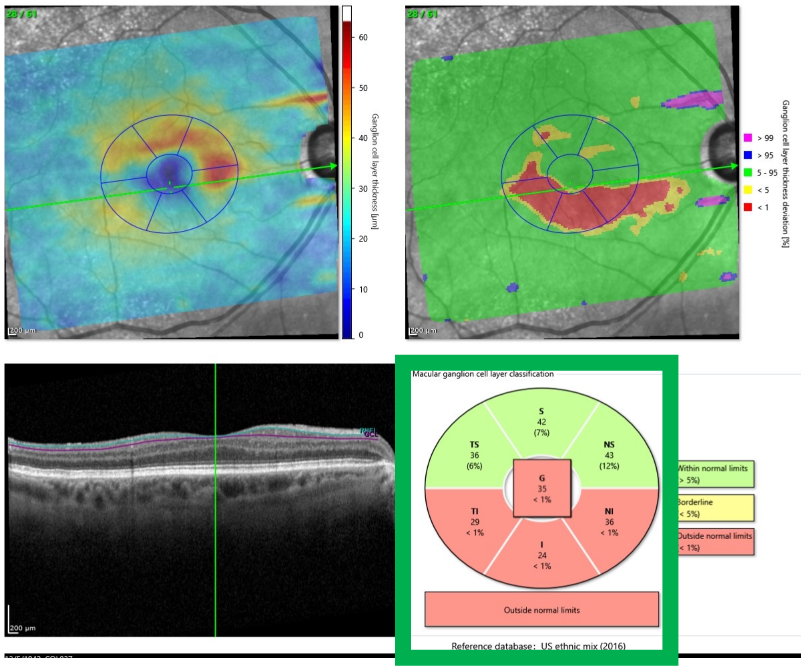

Supplementary Figure 1: An example of the OCT summary metrics that were exported for this study. Red rectangle highlights the Bruch's Membrane Opening – Minimum Rim Width (BMO-MRW) metrics. Blue rectangle highlights the circumpapillary retinal nerve fiber layer (cpRNFL) metrics. Green rectangle highlights the macular ganglion cell layer (GCL) summary metrics.
