# Supplementary material for: Progression of Early Glaucomatous Damage: Performance of Summary Statistics From Optical Coherence Tomography and Perimetry
Source: Transl Vis Sci Technol. 2023 Mar 20;12(3):19. doi: 10.1167/tvst.12.3.19 (PMC10043504; doi:10.1167/tvst.12.3.19)
Supplement: Supplement 2 [file tvst-12-3-19_s002.pdf]

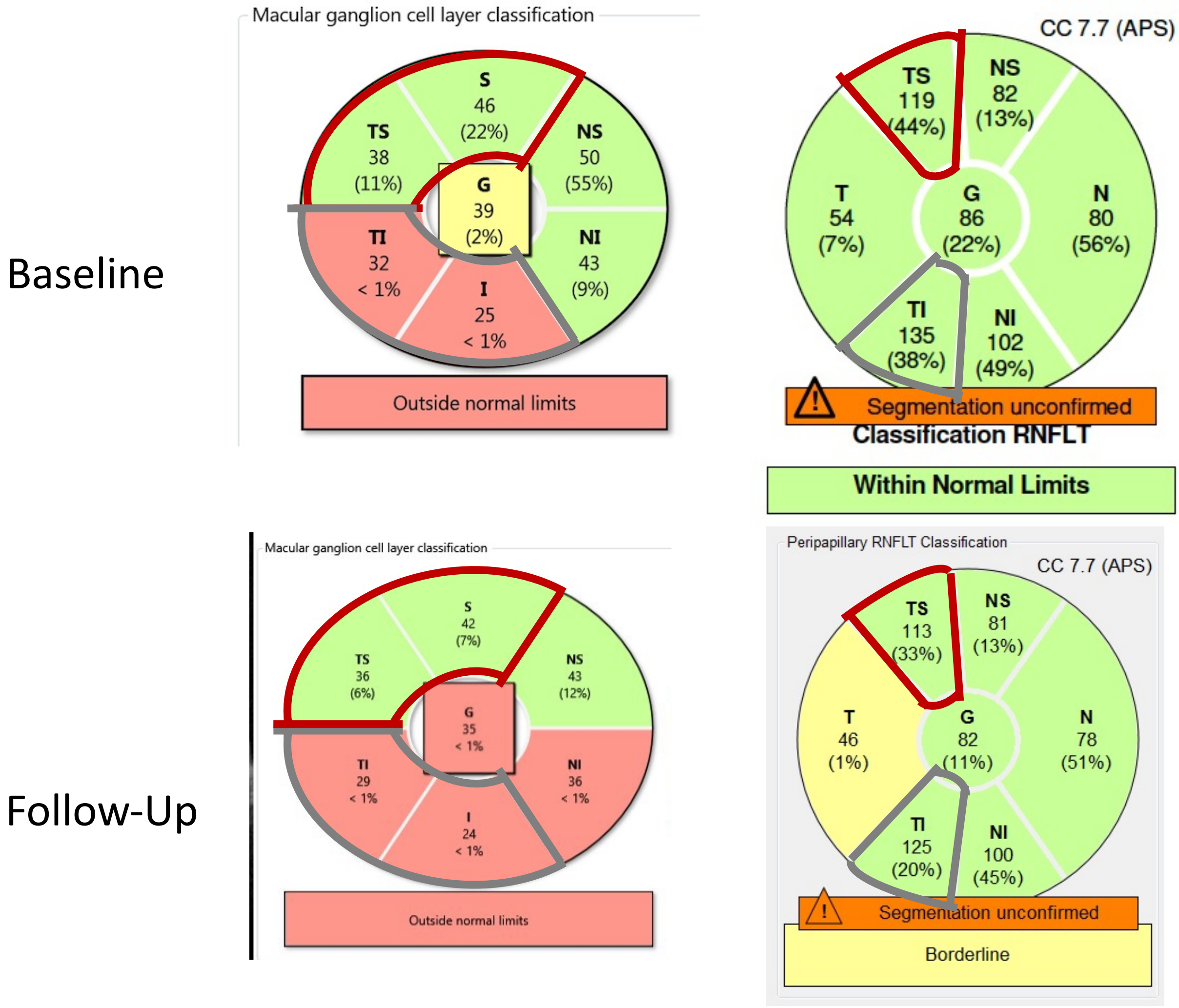

Supplementary Figure 2: The definition of a new Structure-Structure (S-S) summary metric that combines cpRNFL and GCL metrics. The new S-S metric evaluates topographic agreement on the superior (red outlines) or the inferior (grey outlines) hemi-retina. For an eye to be considered progressing, significant thinning must occur inferiorly on the TI cpRNFL sector AND either the TI or the I GCL sector; or, superiorly on the TS cpRNFL sector AND either the TS or the S GCL sector.
