# Supplementary material for: Progression of Early Glaucomatous Damage: Performance of Summary Statistics From Optical Coherence Tomography and Perimetry
Source: Transl Vis Sci Technol. 2023 Mar 20;12(3):19. doi: 10.1167/tvst.12.3.19 (PMC10043504; doi:10.1167/tvst.12.3.19)
Supplement: Supplement 3 [file tvst-12-3-19_s003.pdf]

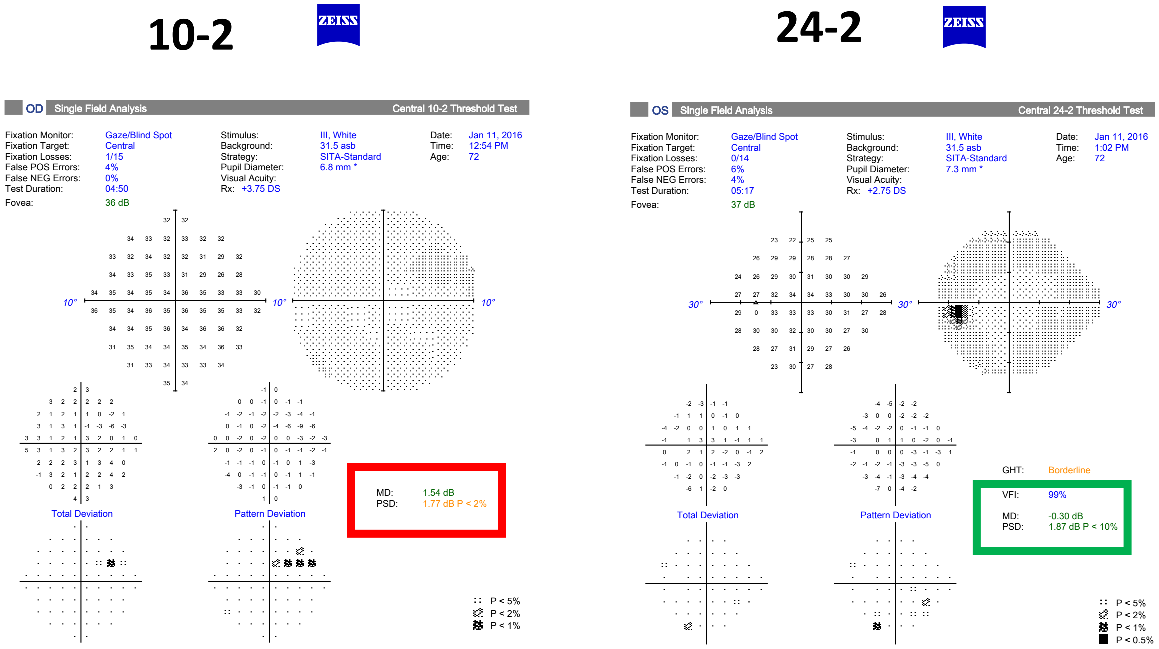

Supplementary Figure 3: An example of the VF summary metrics that were exported for this study. Red rectangle highlights the 10-2 summary metrics. Green rectangle highlights the 24-2 summary metrics.
