# Supplementary material for: Progression of Early Glaucomatous Damage: Performance of Summary Statistics From Optical Coherence Tomography and Perimetry
Source: Transl Vis Sci Technol. 2023 Mar 20;12(3):19. doi: 10.1167/tvst.12.3.19 (PMC10043504; doi:10.1167/tvst.12.3.19)
Supplement: Supplement 4 [file tvst-12-3-19_s004.pdf]

	30 HCs FP (Specificity)	All 91 Patients	17 DP TP (Sensitivity)
cpRNFL metrics - Small circle scan (3.5mm)			
G_{small}	2 (93%)	28	12 (71%)
T_{small}	2 (93%)	25	9 (53%)
TI_{small}	3 (90%)	31	14 (82%)
$\underline{TS_{small}}$	2 (93%)	$\underline{26}$	14 (82%)
N_{small}	2 (93%)	31	7 (41%)
NI_{small}	4 (87%)	21	10 (59%)
NS_{small}	3 (90%)	21	12 (71%)
BMO-MRW metrics – Radial scans			
G_{MRW}	6 (80%)	27	11 (65%)
T_{MRW}	4 (87%)	21	5 (29%)
TI_{MRW}	5 (83%)	19	9 (53%)
TS_{MRW}	3 (90%)	21	10 (59%)
N_{MRW}	8 (73%)	19	9 (53%)
NI_{MRW}	4 (87%)	15	9 (53%)
NS_{MRW}	1 (97%)	20	6 (35%)
GCL Metrics – Posterior Pole Cube scan			
G_{GCL}	2 (93%)	39	14 (82%)
I_{GCL}	1 (97%)	26	12 (71%)
TI_{GCL}	2 (93%)	24	14 (82%)
NI_{GCL}	8 (73%)	37	11 (65%)
S_{GCL}	1 (97%)	12	7 (41%)
TS_{GCL}	1 (97%)	19	7 (41%)
NS_{GCL}	3 (90%)	24	7 (41%)

SUPPLEMENTARY TABLE 1: The number of Statistical Progressors at the 5th percentile cut-off level, as defined by event analysis of OCT summary metrics, are shown for the 30 HC, 91 patients, and the subset of patients categorized as Definite Progressors (DP)
