# Supplementary material for: Progression of Early Glaucomatous Damage: Performance of Summary Statistics From Optical Coherence Tomography and Perimetry
Source: Transl Vis Sci Technol. 2023 Mar 20;12(3):19. doi: 10.1167/tvst.12.3.19 (PMC10043504; doi:10.1167/tvst.12.3.19)
Supplement: Supplement 5 [file tvst-12-3-19_s005.pdf]

| | 30 HCs FP (Specificity) | All 91 Patients | 17 DP TP (Sensitivity) |
|---|---|---|---|
| ***24-2 VF*** | | | |
| MD 24-2 | 2 (93%) | 24 | 9 (53%) |
| supMD 24-2 | 1 (97%) | 15 | 5 (29%) |
| infMD 24-2 | 1 (97%) | 13 | 4 (24%) |
| PSD 24-2 | 2 (93%) | 9 | 4 (24%) |
| VFI 24-2 | 3 (90%) | 16 | 7 (41%) |
| ***10-2 VF*** | | | |
| MD 10-2 | 2 (93%) | 16 | 8 (47%) |
| supMD 10-2 | 0 (100%) | 12 | 4 (24%) |
| infMD 10-2 | 1 (97%) | 11 | 4 (24%) |
| PSD 10-2 | 5 (83%) | 13 | 7 (41%) |

**SUPPLEMENTARY TABLE 2:** The number of Statistical Progressors at the 5$^{th}$ percentile cut-off level, as defined by event analysis of VF summary metrics, are shown for the 30 HC, 91 patients, and the subset of patients categorized as Definite Progressors (DP)
