# Supplementary material for: Progression of Early Glaucomatous Damage: Performance of Summary Statistics From Optical Coherence Tomography and Perimetry
Source: Transl Vis Sci Technol. 2023 Mar 20;12(3):19. doi: 10.1167/tvst.12.3.19 (PMC10043504; doi:10.1167/tvst.12.3.19)
Supplement: Supplement 6 [file tvst-12-3-19_s006.pdf]

	30 HCs<br>FP (Specificity)	All 91 Patients	17 DP<br>TP (Sensitivity)
A. cpRNFL (3.5mm) and BMO-MRW			
G_{small} OR G_{MRW}	7 (77%)	40	15 (88%)
G_{small} AND G_{MRW}	2 (93%)	14	6 (35%)
TI_{small} OR TI_{MRW}	6 (80%)	35	16 (94%)
TI_{small} AND TI_{MRW}	2 (93%)	14	9 (53%)
TS_{small} OR TS_{MRW}	4 (87%)	35	15 (88%)
TS_{small} AND TS_{MRW}	1 (97%)	12	10 (59%)
B. cpRNFL (3.5mm) and GCL			
G AND G_{GCL}	0 (100%)	19	11 (65%)
G OR G_{GCL}	4 (87%)	46	15 (88%)
TI AND (TI_{GCL} OR I_{GCL})<br>[Inferior S-S]	0 (100%)	19	14 (82%)
TI OR (TI_{GCL} OR I_{GCL})	6 (80%)	45	15 (88%)
TS AND (TS_{GCL} OR S_{GCL})<br>[Superior S-S]	0 (100%)	14	10 (59%)
TS OR (TS_{GCL} OR S_{GCL})	3 (90%)	36	15 (88%)
**[Inferior S-S] OR [Superior S-S]**	0 (100%)	22	15 (88%)

SUPPLEMENTARY TABLE 3: The number of Statistical Progressors at the 5th percentile cut-off level, as defined by event analysis on combinations of OCT-OCT summary metrics, are shown for the 30 HC, 91 patients, and the subset of patients categorized as Definite Progressors (DP)
