# Supplementary material for: Progression of Early Glaucomatous Damage: Performance of Summary Statistics From Optical Coherence Tomography and Perimetry
Source: Transl Vis Sci Technol. 2023 Mar 20;12(3):19. doi: 10.1167/tvst.12.3.19 (PMC10043504; doi:10.1167/tvst.12.3.19)
Supplement: Supplement 8 [file tvst-12-3-19_s008.pdf]

	30 HCs<br>FP (Specificity)	All 73 Patients	15 DP<br>TP (Sensitivity)
cpRNFL metrics - Small circle scan (3.5mm)			
G_{small}	5 (83%)	27	13 (87%)
T_{small}	3 (90%)	14	6 (40%)
TI_{small}	3 (90%)	21	12 (80%)
TS_{small}	1 (97%)	14	9 (60%)
N_{small}	4 (87%)	16	5 (33%)
NI_{small}	7 (77%)	23	9 (60%)
NS_{small}	1 (97%)	22	7 (47%)
BMO-MRW metrics – Radial scans			
G_{MRW}	6 (80%)	26	11 (73%)
T_{MRW}	4 (87%)	18	6 (40%)
TI_{MRW}	3 (90%)	27	12 (80%)
TS_{MRW}	5 (83%)	20	10 (67%)
N_{MRW}	5 (83%)	19	11 (73%)
NI_{MRW}	5 (83%)	15	8 (53%)
NS_{MRW}	4 (87%)	18	8 (53%)
GCL Metrics – Posterior Pole Cube scan			
G_{GCL}	7 (77%)	29	12 (80%)
I_{GCL}	3 (90%)	17	10 (67%)
TI_{GCL}	2 (93%)	20	12 (80%)
NI_{GCL}	6 (80%)	23	7 (47%)
S_{GCL}	6 (80%)	18	7 (47%)
TS_{GCL}	2 (93%)	16	8 (53%)
NS_{GCL}	3 (90%)	19	7 (47%)

SUPPLEMENTARY TABLE 5: The number of Statistical Progressors at the 2.5th percentile cut-off level, as defined by trend analysis of OCT summary metrics, are shown for the 30 HC, 73 patients, and the subset of patients categorized as Definite Progressors (DP)
