# Supplementary material for: Progression of Early Glaucomatous Damage: Performance of Summary Statistics From Optical Coherence Tomography and Perimetry
Source: Transl Vis Sci Technol. 2023 Mar 20;12(3):19. doi: 10.1167/tvst.12.3.19 (PMC10043504; doi:10.1167/tvst.12.3.19)
Supplement: Supplement 9 [file tvst-12-3-19_s009.pdf]

	30 HCs<br>FP (Specificity)	All 73 Patients	15 DP<br>TP (Sensitivity)
24-2 VF			
MD 24-2	3 (90%)	18	9 (60%)
supMD 24-2	1 (97%)	12	5 (33%)
infMD 24-2	2 (93%)	8	4 (27%)
PSD 24-2	2 (93%)	6	4 (27%)
VFI 24-2	2 (93%)	13	7 (47%)
10-2 VF			
MD 10-2	3 (90%)	13	6 (40%)
supMD 10-2	0 (100%)	12	4 (27%)
infMD 10-2	1 (97%)	11	4 (27%)
PSD 10-2	5 (83%)	11	4 (27%)

SUPPLEMENTARY TABLE 6: The number of Statistical Progressors at the 2.5th percentile cut-off level, as defined by trend analysis of VF summary metrics, are shown for the 30 HC, 73 patients, and the subset of patients categorized as Definite Progressors (DP)
