# Supplementary material for: Progression of Early Glaucomatous Damage: Performance of Summary Statistics From Optical Coherence Tomography and Perimetry
Source: Transl Vis Sci Technol. 2023 Mar 20;12(3):19. doi: 10.1167/tvst.12.3.19 (PMC10043504; doi:10.1167/tvst.12.3.19)
Supplement: Supplement 10 [file tvst-12-3-19_s010.pdf]

| | 30 HCs<br>FP (Specificity) | All 73 Patients | 15 DP<br>TP (Sensitivity) |
|---|---|---|---|
| ***A. cpRNFL (3.5mm) and BMO-MRW*** | | | |
| $G_{small}$ OR $G_{MRW}$ | 9 (70%) | 35 | 14 (93%) |
| $G_{small}$ AND $G_{MRW}$ | 2 (93%) | 18 | 10 (67%) |
| $TI_{small}$ OR $TI_{MRW}$ | 5 (83%) | 34 | 15 (100%) |
| $TI_{small}$ AND $TI_{MRW}$ | 1 (97%) | 14 | 9 (53%) |
| $TS_{small}$ OR $TS_{MRW}$ | 5 (83%) | 25 | 11 (73%) |
| $TS_{small}$ AND $TS_{MRW}$ | 1 (97%) | 9 | 8 (53%) |
| ***B. cpRNFL (3.5mm) and GCL*** | | | |
| G AND $G_{GCL}$ | 1 (97%) | 18 | 11 (73%) |
| G OR $G_{GCL}$ | 11 (63%) | 36 | 13 (87%) |
| TI AND ($TI_{GCL}$ OR $I_{GCL}$)<br>[Inferior S-S] | 1 (97%) | 15 | 12 (80%) |
| TI OR ($TI_{GCL}$ OR $I_{GCL}$) | 7 (77%) | 32 | 14 (93%) |
| TS AND ($TS_{GCL}$ OR $S_{GCL}$)<br>[Superior S-S] | 0 (100%) | 6 | 5 (33%) |
| TS OR ($TS_{GCL}$ OR $S_{GCL}$) | 8 (74%) | 30 | 12 (80%) |
| **[Inferior S-S] OR [Superior S-S]** | 0 (100%) | 16 | 12 (80%) |

**SUPPLEMENTARY TABLE 7:** The number of Statistical Progressors at the 2.5$^{th}$ percentile cut-off level, as defined by trend analysis on combinations of OCT-OCT summary metrics, are shown for the 30 HC, 73 patients, and the subset of patients categorized as Definite Progressors (DP)
