# Supplementary material for: Progression of Early Glaucomatous Damage: Performance of Summary Statistics From Optical Coherence Tomography and Perimetry
Source: Transl Vis Sci Technol. 2023 Mar 20;12(3):19. doi: 10.1167/tvst.12.3.19 (PMC10043504; doi:10.1167/tvst.12.3.19)
Supplement: Supplement 11 [file tvst-12-3-19_s011.pdf]

	30 HCs FP (Specificity)	All 73 Patients	15 DP TP (Sensitivity)
Structure and Function			
G_{small} AND MD 24-2	1 (97%)	14	8 (47%)
G_{small} OR MD 24-2	6 (80%)	44	13 (87%)
G_{GCL} AND MD 10-2	0 (100%)	16	9 (53%)
G_{GCL} OR MD 10-2	6 (80%)	39	14 (93%)
**[G_{small} OR G_{GCL}] AND [MD_{24} OR MD_{10}]**	2 (93%)	23	11 (73%)
[G_{small} AND G_{GCL}] AND [MD_{24} AND MD_{10}]	0 (100%)	9	4 (27%)
**[Inferior S-S] OR [Superior S-S] AND [MD_{24} OR MD_{10}]**	1 (97%)	11	11 (73%)
**[Inferior S-S] OR [Superior S-S] AND [$secMD_{24}$ OR $secMD_{10}$]**	1 (97%)	11	11 (73%)

SUPPLEMENTARY TABLE 8: The number of Statistical Progressors at the 2.5th percentile cut-off level, as defined by trend analysis on combinations of OCT-VF summary metrics, are shown for the 30 HC, 73 patients, and the subset of patients categorized as Definite Progressors (DP)
